# Increased frontal functional connectivity correlates with structural disconnection in the anterior corpus callosum in healthy older adults

**DOI:** 10.1038/s41598-025-19143-y

**Published:** 2025-09-12

**Authors:** Hanna Braaß, Nadine Reiter, Winifried Backhaus, Paweł P. Wróbel, Fanny Quandt, Robert Schulz, Christian Gerloff, Focko L. Higgen

**Affiliations:** 1 Department of Neurology, University Medical Center Hamburg-Eppendorf, Martinistraße 52, 20246 Hamburg, Germany; 2 Department of Systems Neuroscience, University Medical Center Hamburg- Eppendorf, Martinistraße 52, 20246 Hamburg, Germany; 3 Department of Psychiatry and Psychotherapy, University Medical Center Hamburg- Eppendorf, Martinistraße 52, 20246 Hamburg, Germany

**Keywords:** RsfMRI, DTI, Tractography, Healthy aging, Corpus callosum, Cognitive ageing, Neural ageing, Neurology

## Abstract

Cognitive aging is a heterogeneous process characterized by a varied decline in brain performance. While the neurophysiological basis of this decline remains poorly understood, age-related alterations in structural connectivity (SC) and functional connectivity (FC) have been implicated. Understanding the relationship between these changes is crucial for identifying early markers of cognitive impairment. In our previous work, we observed that in healthy older adults, diminished performance in a tactile recognition task was associated with reduced fractional anisotropy (FA) in the anterior corpus callosum (aCC). The aim of this study was to investigate the impact of this age-related SC degeneration on the FC between the connected regions. In the current analysis, we examined resting-state functional MRI data from 29 healthy older adults, who were categorized into two subgroups - high and low performers - based on their performance in a tactile recognition task and a younger control group (N = 20). We conducted a region of interest (ROI) to ROI analysis to evaluate FC between frontal regions connected via the aCC. Subsequently, we compared the FC between the groups and assessed its correlation with FA values in the aCC across all older participants based on a TBSS analysis from our previous work. We found that poorer performance in a tactile recognition task was associated with increased FC between frontal regions. The second main finding was a negative correlation between FA in the aCC and FC between frontal regions. Our findings suggest that structural degradation of the underlying pathways may initially lead to increased FC within the connected regions, possibly as a compensatory mechanism to preserve cognitive function. However, as no direct brain–behavior correlation was assessed, this interpretation remains tentative. The aCC appears to be particularly vulnerable to age-related structural decline, which may underlie functional changes in frontal regions observed with aging.

**Supplementary Information:**

The online version contains supplementary material available at 10.1038/s41598-025-19143-y.

## Introduction

Cognitive aging is a heterogeneous process marked by interindividual variability in the extent and pattern of functional decline^1^. While proteomic analyses have identified initial candidate biomarkers for predicting the development of dementia^2^the underlying neurophysiological mechanisms remain poorly understood. In particular, the physiological significance of observed structural and functional brain alterations has yet to be elucidated^3,4^. To gain deeper insights into cognitive aging, it is essential to clarify the relationships between the various age-related structural and functional changes and their behavioral consequences. This understanding could lead to identifying early markers of cognitive decline, thus enabling optimal support for affected older individuals^5–7^.

One common finding with increasing age is a widespread decrease of structural connectivity (SC) across the brain with a pronounced focus on the frontal lobe^8–10^. SC is calculated from fractional anisotropy (FA), derived from diffusion-tensor imaging (DTI). Within the limitations of fiber-tracking, FA is commonly interpreted as reflecting white matter integrity^11,12^. However, it represents a compound measure influenced by several microstructural features, such as axonal density, myelination, and fiber coherence^13^. Numerous imaging studies have been conducted to examine age-related changes in SC^14,15^ and to differentiate between changes related to healthy aging and those associated with neurological diseases such as dementia^16,17^ or other neurodegenerative conditions like Parkinsonian disease^18^. ’Cortical disconnection’ has repeatedly been reported to be a significant factor in age-related cognitive decline^19^.

To fully comprehend the relevance of structural disconnection, it is crucial to understand its functional consequences. The effects of decreased SC on functional connectivity (FC) are not yet fully understood. Functional brain connectivity is often assessed through the analysis of co-activations of hemodynamic signals measured by blood-oxygen-level-dependent (BOLD) fMRI^9^. Since SC provides the foundational scaffolding for FC, age-related structural disconnection is expected to affect FC between brain regions^20,21^. However, the data on this relationship are ambiguous. Several studies suggest that SC and FC change largely independently with age^22–24^. Other research points to a closer relationship, mainly indicating that structural disconnection leads to reduced FC^25–27^. However, the relationship between SC and FC does not seem straightforward, as evidenced by resting-state functional MRI (rsfMRI), a common technique for assessing connectivity in brain networks during task-free fMRI^28,29^. An increase in FC within resting-state networks during less severe stages of SC decrease has been reported^30^. Interestingly, studies on patients with different neurological diseases suggest a similar FC-SC relationship. In Alzheimer’s disease, an increase in default mode network FC has been observed during the initial decline in SC^31,32^ and in multiple sclerosis, cortical networks exhibit increased FC despite decreased SC, with this increase correlating with the severity of cognitive impairment^33^. Thus, alterations in SC and FC might contribute to age-related cognitive decline, but the precise relationship between these metrics and the mechanisms differentiating healthy from non-healthy aging remains unclear.

In a previous report, we explored the relationship between microstructural white matter characteristics (FA) and sensory processing in healthy older adults^34^. Sensory processing is a promising entry point for understanding mechanisms of brain aging. Age-related impairments affect all sensory modalities, e.g. visual acuity and auditory and tactile thresholds, and substantially impact independence and activities of daily living^35–38^. Importantly, a decline in sensory acuity has repeatedly been shown to predict subsequent decline in higher cognitive functions, suggesting that sensory degradation may play a causal role in cognitive aging^39–42^. We found a significantly reduced FA of transcallosal fibers in the anterior part of the corpus callosum (aCC) in a group of older participants with poor performance in a complex tactile recognition task compared to a higher performing group. The aCC connects various regions of the bilateral (pre-)frontal cortex and has been shown to exhibit disproportionate age-related microstructural decline compared to other callosal and association tracts^8,15^suggesting it is particularly susceptible to aging effects. Our findings suggest that structural disconnection in frontal networks critical for top-down control of sensory processing may contribute to cognitive decline with aging^43^. Identifying clearly defined SC degeneration provides a foundation for investigating the effect of SC alterations on FC. No previous study has specifically linked anterior callosal microstructure with resting-state frontal FC in relation to tactile recognition performance, a key marker of top-down sensory integration in aging.

To this end, in the current study, we used rsfMRI data acquired in the same study. We examined whether differences in performance on the complex tactile recognition task were also evident in resting-state FC and whether the reported SC changes in the aCC were associated with the FC between (pre-)frontal regions. We performed three different analyses. The first analysis compares whole and frontal brain FC between a younger control group and older high- and low-performers. The second analysis compares frontal FC between older high- and low-performers. In the third analysis, FA values from the aCC, as reported previously, were correlated with the FC values over the entire group of older participants.

We hypothesized (i) that the group of older low performers would exhibit increased frontal FC compared to the group of older high performers, consistent with prior findings on early structural and cognitive decline, and (ii) that lower FA in the aCC would be associated with increased FC between connected frontal regions across all older adults.

## Materials and methods

### Participants

All 29 older participants (16 female, mean age 72.7 years, range 65–82 years) and 20 younger participants (11 female, mean age 24.1, range 20–28) from our previous study were re-analyzed^34^. All participants were right-handed according to the Edinburgh handedness inventory^44^had normal or corrected to normal vision, no history or symptoms of neuropsychiatric disorders, and no recent history of medication that affects the nervous system. As in our previous study, the older participants were grouped into two groups based on performance in a tactile recognition task (see below): 10 O-LP (older-low-performers, 5 female, mean age 74.1 years, range 68–82 years) and 19 O-HP (older-high-performers, 11 female, mean age 71.9 years, range 65–79 years). For further information on the participants and groups, see Table 1. All participants received monetary compensation.

## Ethics statement

The study was conducted following the Declaration of Helsinki and approved by the local ethics committee of the Medical Association of Hamburg (PV5085). All participants gave written informed consent.

## Clinical and behavioral assessment

Participants underwent an assessment procedure that included a neurological examination, the Mini-Mental State (MMSE, cut-off ≥ 28)^45^the DemTect (cut-off ≥ 13)^46^a 2-point-discrimination test (cut-off > 3 mm)^47^a test of the mechanical detection threshold (MDT, v. Frey Filaments, OptiHair2-Set, Marstock Nervtest, Germany, cut-off > 0.75mN)^48^ and the subjectively experienced attention deficits with a standardized questionnaire (“Fragebogen erlebter Defizite der Aufmerksamkeit” FEDA A, B, C)^49^.

## Task design and grouping

In the original study, participants performed a tactile recognition task, representing a delayed match-to-sample task with steps of increasing complexity. The tactile presentation was performed with a Braille stimulator (QuaeroSys Medical Devices, Schotten, Germany), with the fingertip of the right index finger placed above the stimulating unit. The stimuli consisted of four geometric patterns, each of them formed by four dots. For pattern presentation a stimulus was chosen pseudo-randomly from the stimulus set. During the response period, all four patterns were presented visually on the screen, and participants were asked to indicate which of the four patterns had been presented. The experiment began with a very simple pattern set and a stimulation time of 800ms to get the participants acquainted with the tactile stimulation. After a minimum of five blocks, each one consisting of 16 trials and accuracy of at least 75% in three of five consecutive blocks, participants could proceed to the next step. If participants did not reach the target accuracy within 15 blocks, they were excluded from further participation. In the next step the stimulus set consisted of four more complex patterns. To train participants in the recognition of the target patterns, stimulation occurred again with a long stimulation time of 800ms. If again, participants were able to recognize patterns with the previously defined accuracy, the stimulation time of the target patterns was decreased to 500ms as a final step of the recognition task. The participants who finally reached the predefined performance level were categorized as “older-high-performers” (O-HP, *n* = 19), while those who did not reach the predefined accuracy were classified as “older-low-performers” (O-LP, *n* = 10). We grouped all participants not reaching the predefined accuracy target at one of the steps of the tactile recognition task together, as they all did not show any deficits in the initial assessment, however a clear performance difference in tactile recognition compared to the younger participants and O-HP. O-HP achieved ≥ 75% correct responses in all three blocks of the tactile recognition task (mean accuracy = 81.4%, SD = 4.5), while O-LP participants fell below the 75% threshold in at least one block and were consequently excluded from further participation (mean accuracy = 52.2%, SD = 3.8). Reaction times were not collected. For full technical details see^34^.

## MRI data acquisition

A 3 T Skyra MRI scanner (Siemens Healthineers, Erlangen, Germany) and a 32-channel head coil were used to acquire multimodal imaging data, including structural high-resolution T1-weighted images, rsfMRI images, and diffusion-weighted images. For the T1-weighted sequence, a three-dimensional magnetization-prepared rapid gradient echo (3D-MPRAGE) sequence was used with the following parameters: repetition time (TR) = 2500 ms, echo time (TE) = 2.12 ms, flip angle 9°, 256 coronal slices with a voxel size of 0.8 × 0.8 × 0.9 mm³, field of view (FOV) = 240 mm. The parameters of rsfMRI were: FOV = 260 mm, TR = 2 s, TE = 30 ms, a 72 × 72 × 32 matrix, voxel size of 3 × 3 × 3 mm³, flip angle 90°, and 210 images. Before the resting-state scans, the participants were asked to focus on a black cross located behind the scanner, which could be viewed via a mirror. For the diffusion-weighted sequence, a spin-echo, echo-planar imaging (EPI) sequence was applied with the following parameters: TE = 82 ms, TR = 10,000 ms, flip angle = 90°, matrix size = 104 × 128 matrices, FOV = 208 × 256 mm^2^ voxel size of 2.0 × 2.0 × 2.0 mm³, partial Fourier factor = 0.75, 75 contiguous transversal slices, one image with b = 0 s/mm2, 64 images with b = 1500 s/mm^2^ (64 non-collinear directions), one single phase encoding direction.

### Image processing

The default pre-processing pipeline for volume-based analyses within the CONN-toolbox v20.b (SPM 12) was used to preprocess the rsfMRI data^50^. The first ten volumes were discarded to account for magnetization equilibrium effects. During the initial preprocessing, all functional images were realigned (motion corrected), centered, slice time corrected, corrected for motion artifacts using the artifact detection tools (ART), and coregistered to their corresponding T1-weighted images.

Images were identified as outliers if the head movement was more than five standard deviations from the mean intensity of the entire run or outside a 97th percentile threshold. All structural images were then centered and segmented into CSF (cerebrospinal fluid), grey and white matter, and spatially normalized to the Montreal Neurological Institute (MNI) template. Functional images were normalized to MNI space using the deformation field from the corresponding structural images and spatially smoothed to allow for better registration and reduction of noise using a 6 mm full width at half maximum (FWHM) Gaussian kernel. After preprocessing, motion parameters were derived from rigid-body realignment and their derivatives. Five potential noise components (average BOLD signal and the first four components in a PCA (principle component analysis) of the covariance within the subspace orthogonal to the average BOLD signal) derived from CSF and white matter using the aCompCor (anatomical component-based noise correction) procedure, were regressed from the signal^51,52^. Global signal regression was not included in the analysis to avoid potential false anti-correlations^53^. A temporal band-pass filter between 0.008 and 0.1 Hz was applied to focus on slow-frequency fluctuations while minimizing the influence of physiological signals, head motion, and other noise sources^54^. The preprocessing and Tract-based spatial statistic (TBSS) analysis of the diffusion-weighted images are described in detail in^34^ and are not part of this analysis. In short, a TBSS analysis was performed to compare the diffusion-weighted images of O-LP and O-HP. The result was a significantly reduced FA of transcallosal fibers in the anterior corpus callosum (aCC), in O-LP compared to O-HP. The individual mean FA values (for O-LP and O-HP) in this area of the aCC were extracted for further analysis. The FA values were not extracted for the younger participants. As reported in our previous study, the TBSS analysis revealed significantly higher FA across nearly all white matter regions, including the anterior corpus callosum, in younger participants compared to both older groups^34^.

## ROI – ROI analyses

**Fig. 1:**
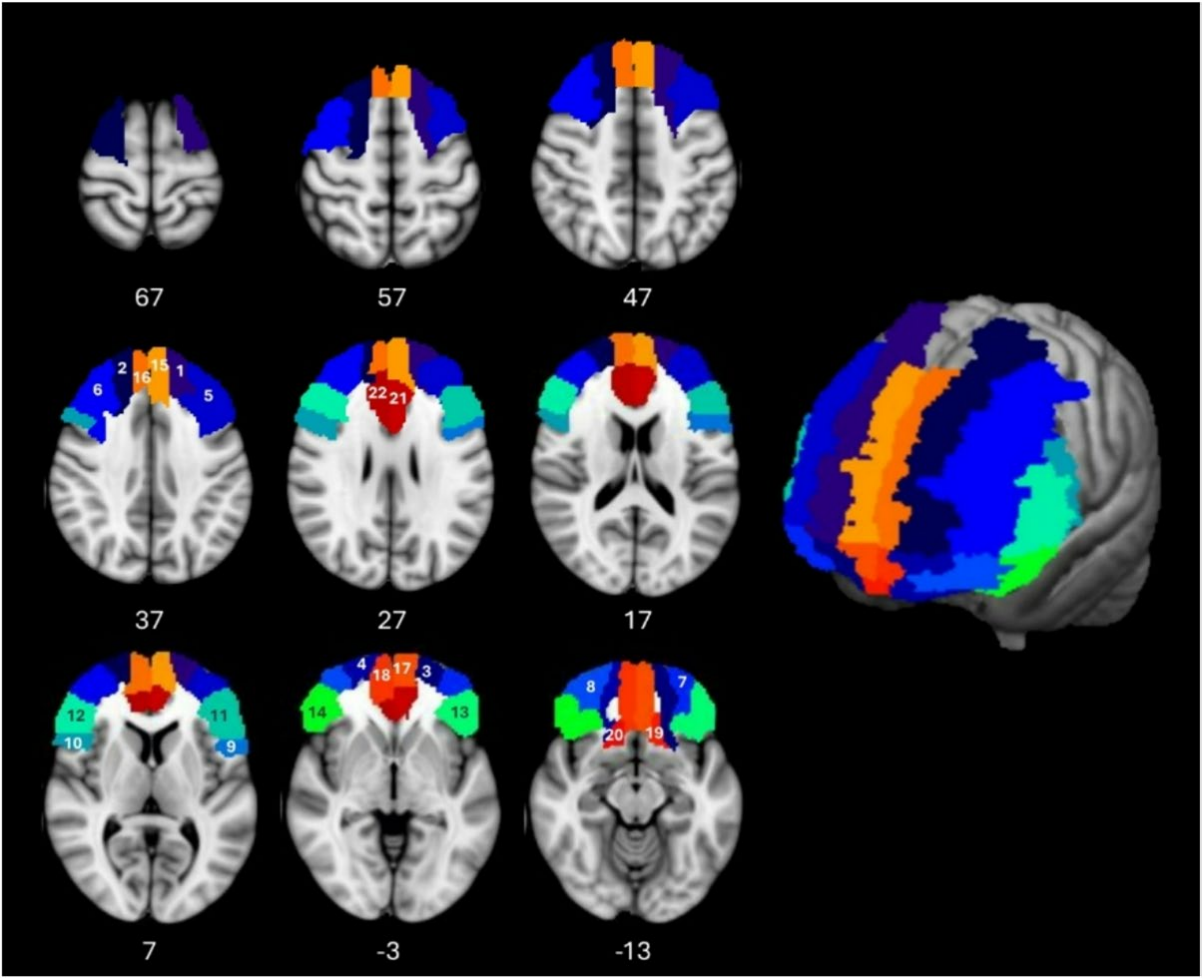
Schematic overview of the AAL ROIs (Table S1) used 1,2, Frontal Sup. L/R, 3,4 Front. Sup. Orb. L/R, 5,6 Frontal Mid L/R, 7,8 Frontal Mid. Orb. L/R, 9/10 Frontal Inf. Oper. L/R, 11,12 Frontal Inf. Tri. L/R, 13,14 Frontal Inf. Orb. L/R, 15,16 Frontal Sup. Med L/R, 17,18 Frontal Med. Orb. L/R, 19,20 Rectus L/R, 21,22 Cingulum Ant. L/R.The ROIs are projected onto the MNI152_T1_1mm template, L = Left, R = Right, slice numbers are MNI z-coordinates.

For the first ROI-to-ROI analysis, all 116 ROIs from the AAL atlas^55^ were chosen as regions of interest (ROIs). For the second, the *frontal* ROI-to-ROI analysis, 22 frontal areas that have been shown to be connected via the aCC were extracted from the AAL atlas as ROIs (Fig. 1). The ROIs were chosen based on the tractography analysis from the aCC in our previous work^34^ and reports in the literature^56,57^. The selected ROIs are listed in Table S1, Fig. 1. For the analysis, the mean BOLD signal time course was extracted from every ROI after preprocessing. In the first level analysis, the Fisher-transformed bivariate correlation coefficients (= FC values) between each pair of ROI-ROI connections were calculated for each participant and used for further analysis.

## Statistical analyses

### Assessment

As described in^34^ to test the group differences (O-LP vs. O-HP and Young vs. O-LP and O-HP), a linear model was defined utilizing R’s lm command to investigate the relationship between the assessment variables Age, MDT, 2-point-discrimination, MMSE, DemTect, FEDA (A, B, C) as dependent variables and GROUP as an independent variable. Age was included in the model to test for age differences in the groups O-HP and O-LP. The comparison was performed using lsmeans (R-package: lsmeans) and pairwise comparison between the resulting contrasts. Benjamini-Yekutieli (BY) was used to adjust for multiple comparisons. For post-hoc testing, a MANOVA was used with GROUP as an independent variable and BY correction to adjust for multiple comparisons.

### Comparison between younger and older participants

For the initial group comparison between younger and older participants, two analyses were performed across all AAL ROIs to examine whole-brain differences in FC: one comparing younger participants (Y) to O-LP, and another comparing Y to O-HP. Additionally, a group comparison was conducted focusing specifically on the frontal AAL regions, comparing Y with both and O-LP and O-HP.

### Comparison between O-LP and O-HP and correlation between FC and FA

For the second group comparisons, the two older groups (O-LP and O-HP) were compared for all *frontal* ROI-ROI connections.

In an additional second-level analysis, a correlation between FC and FA values was calculated. For this analysis, the FC values were correlated with the individual mean FA of the aCC for all 29 participants. The FA values were the result of our previously published analysis^34^ as described above.

For the group comparisons and correlation analysis, the Functional Network Connectivity (FNC) multivariate parametric statistics, an analysis with a data-driven hierarchical clustering procedure^58^was used with an FDR-corrected connection threshold of *p* < 0.05 and an FDR-corrected cluster threshold of *p* < 0.05.

## Results

### Participants

All 29 older participants (16 female, mean age 72.7 years, range 65–82 years) and 20 younger participants (11 female, mean age 24.1, range 20–28) were included in the final analyses. The whole group of older participants was further divided into 2 groups based on performance in the described tactile recognition task: 10 O-LP (5 female, mean age 74.1 years, range 68–82 years) and 19 O-HP (11 female, mean age 71.9 years, range 65–79 years).

### Assessment

**Table 1:** Assessment and mean FA in the aCC.

Metrics	Y (*n* = 20)	O (*n* = 29)	O-LP (*n* = 10)	O-HP (*n* = 19)
**Age**	24.1 (± 2.6)^+#^	72.7 (± 4.3)	74.1 (± 3.9)^+^	71.9 (± 4.4)^#^
**Education (years)**	12.5 (± 0.6)	11.0 (± 1.8)	11.7 (± 2.0)	10.7 (± 1.6)
**DemTect**	17.8 (± 0.6)^#^	16.3 (± 1.6)	16.9 (± 1.6)	16.0 (± 1.6)^#^
**MMSE**	29.7 (± 0.6)	29.4 (± 0.7)	29.2 (± 0.8)	29.5 (± 0.6)
**2-Point (mm)**	2.1 (± 0.2)	2.3 (± 0.5)	2.4 (± 0.5)	2.2 (± 0.4)
**MDT (mN)**	0.28 (± 0.1)^+^	0.59 (± 0.5)	0.65 (± 0.4)^+^	0.56 (± 0.5)
**FEDA A**	4.28 (± 0.4)	4.23 (± 0.5)	4 (± 0.5)	4.35 (± 0.4)
**FEDA B**	4.55 (± 0.4)	4.42 (± 0.6)	3.97 (± 0.8)	4.65 (± 0.4)
**FEDA C**	4.35 (± 0.6)	4.16 (± 0.7)	3.75 (± 0.9)	4.38 (± 0.5)
**Mean FA**	-	0.62 (± 0.05)	0.58 (± 0.05) *	0.65 (± 0.02) *

The assessment data of the groups of included younger (= Y) and older participants (= O) is shown and additionally divided into two groups, older-low-performers (= O-LP) and older-high-performers (= O-HP). Mean values are shown ± standard deviation.* The mean FA-value in the aCC showed a significant difference between O-LP and O-HP (two-sided unpaired t-test, p < 0.001). The table is a modified version of a table in^34^. + Indicate significant differences between Young and O-LP (p-values ≤ 0.01). # Indicate significant differences between Young and O-HP (p-values ≤ 0.01). No significant behavioral differences between O-LP and O-HP were observed except for FA. Mini-Mental State (MMSE), mechanical detection threshold (MDT), “Fragebogen erlebter Defizite der Aufmerksamkeit” (FEDA), fractional anisotropy (FA).

Table 1 presents the results of the clinical and behavioral assessment of the different groups and the mean FA in the aCC of the older participants. There was a significant difference in FA in the aCC between O-LP and O-HP, but no significant differences in the assessments.

### Group comparisons of FC

#### Comparison between younger and older participants

**Fig. 2:**
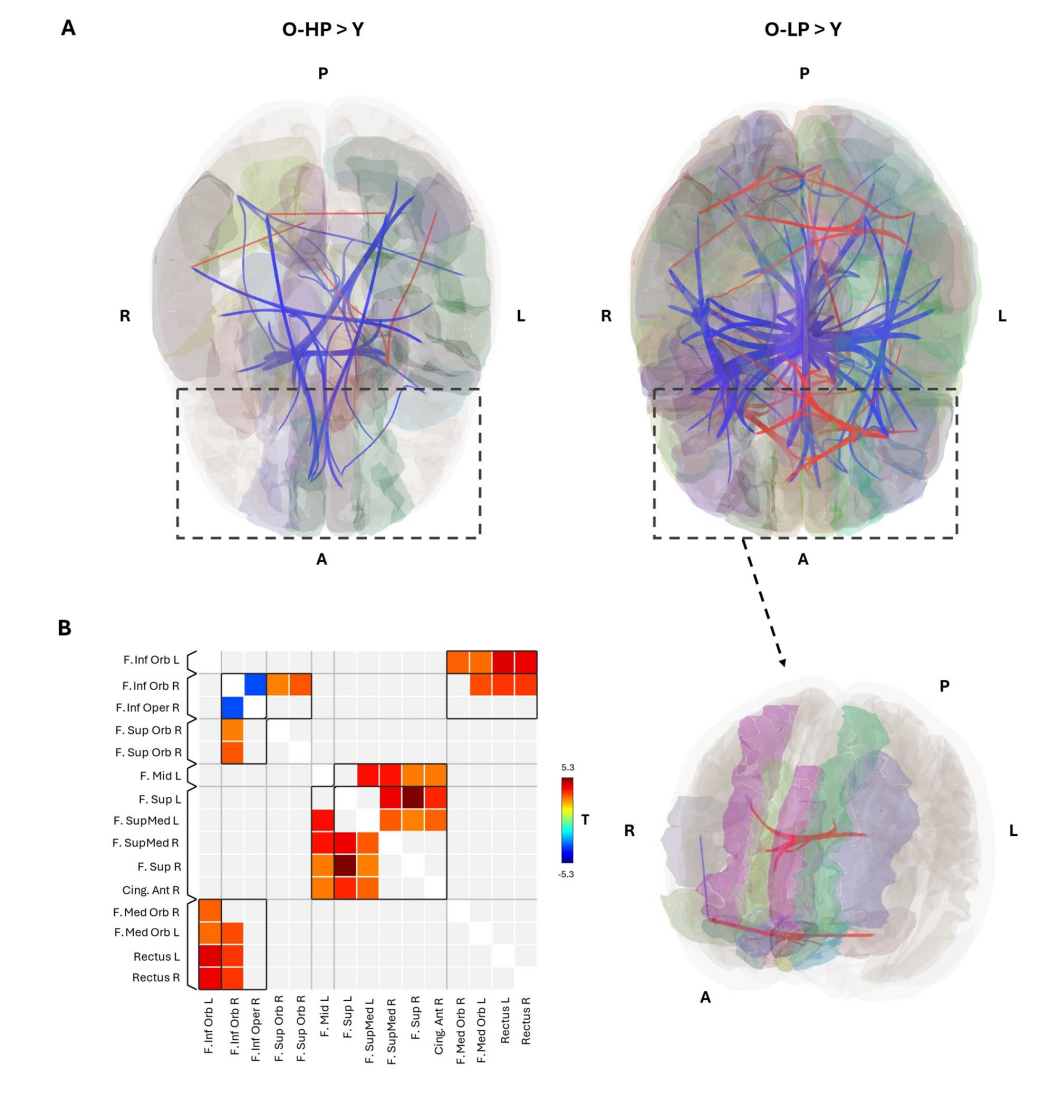
Comparison of FC between Y and O-HP and Y and O-LP. **A** Comparison of FC between all AAL ROIs between Y and O-HP (left) and Y and O-LP (right) shows statistically significantly higher FC in O-LP in red and lower FC in O-LP in blue. **B** The colored matrix displays the T-values of significant differences between the FC of the Y and O-LP sorted along significant clusters from FNC statistics; red/yellow: higher FC in the O-LP group; R = Right, L = Left, A = Anterior, P = Posterior, F = Frontal, Sup = Superior, Med = Medial, Mid = Middle, Orb = Orbital, Inf = Inferior, T = T-value.

Group comparisons of FC across all AAL-ROIs revealed several ROI-to-ROI connections exhibiting significantly greater FC in Y compared to both O-HP and O-LP (Fig. 2A). These encompassed both intra- and interhemispheric connections. Notably, the comparison between Y and O-LP yielded a greater number of significantly enhanced connections than the comparison between Y and O-HP, a finding that may be partially attributed to the smaller sample size of O-LP (*n* = 10) relative to O-HP (*n* = 19).

In addition, some ROI-to-ROI connections demonstrated higher FC in both O-HP and O-LP, particularly between posterior ROIs. Only in the comparison between Y and O-LP elevated FC between frontal regions was observed.

This pattern was further corroborated by the targeted analysis of frontal AAL ROIs., which revealed significantly increased FC among several frontal connections, which particularly encompass connections to parts of the Inferior frontal gyrus (IFG), Middle frontal gyrus (MFG), Superior frontal gyrus (SFG), gyrus rectus (GR), and anterior part of the cingulate gyrus (ACG) in O-LP (Fig. 2B, Table S2), while no significant differences were observed between Y and O-HP in the same regions.

### Comparison between O-HP and O-LP

**Fig. 3:**
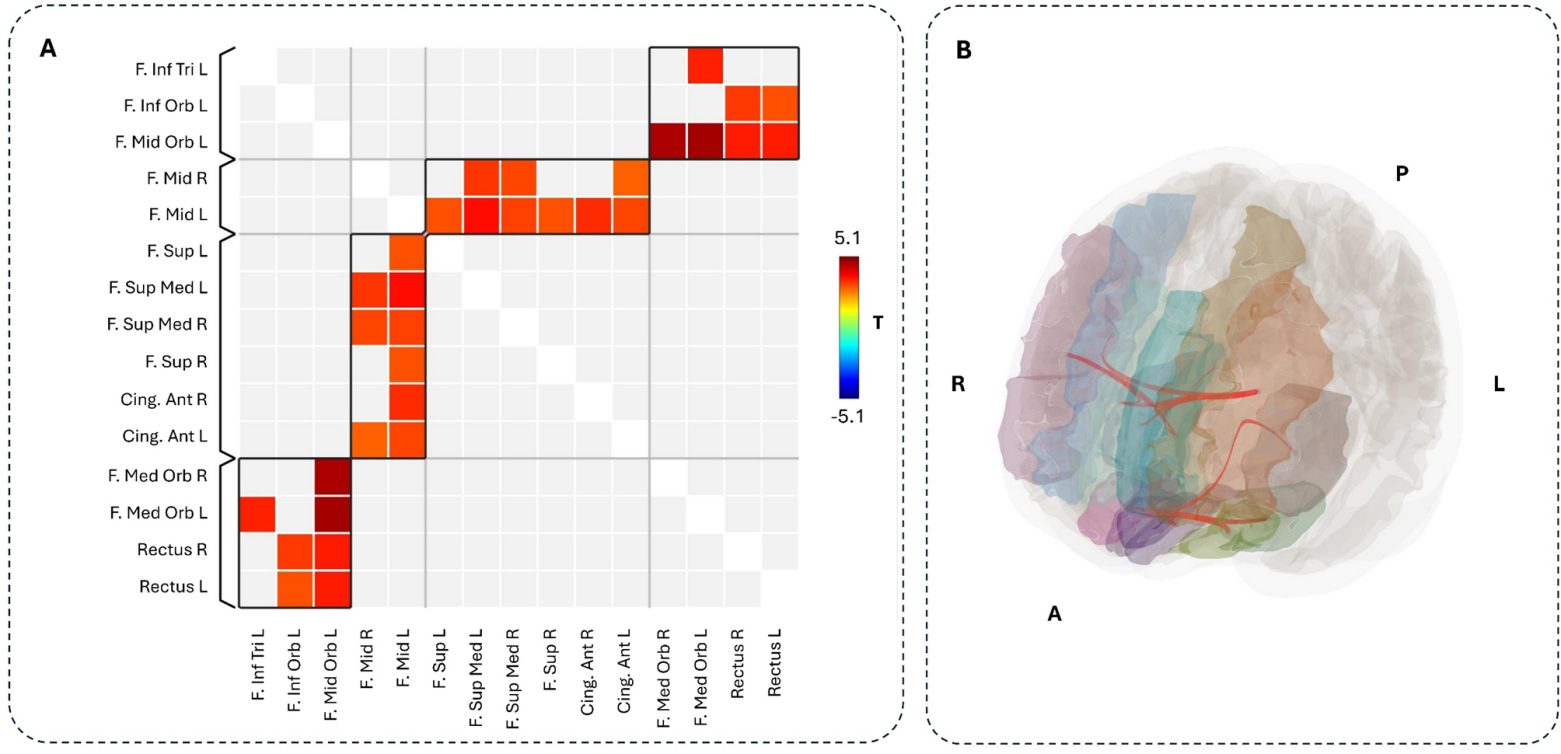
Comparison of frontal FC between the two groups, FC(O-LP) > FC(O-HP). **A** The colored matrix displays the T-values of significant differences between the FC of the O-LP and O-HP sorted along significant clusters from FNC statistics; red/yellow: higher FC in the O-LP group; **B** The ROIs, which show a significantly higher connection, are highlighted in different colors; statistically significant increased FC in O-HP compared to O-LP are shown in red; R = Right, L = Left, A = Anterior, P = Posterior, F = Frontal, Sup = Superior, Med = Medial, Mid = Middle, Orb = Orbital, Inf = Inferior, T = T-value.

**Fig. 4:**
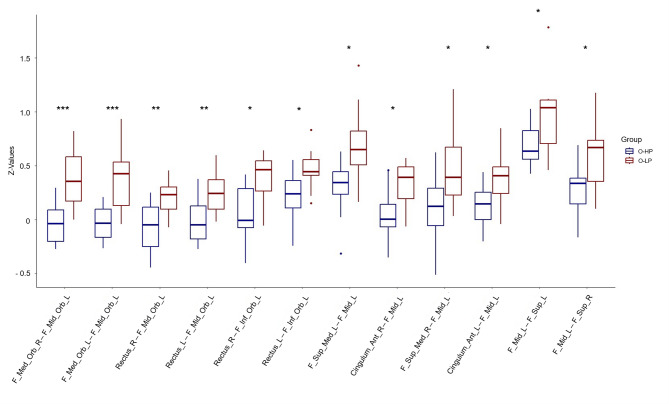
Representation of the distribution of the Z-Values as measurement of the FC of the frontal connections, which exhibit both a significant group difference and a significant correlation with the FA values. The Z values of the two groups are displayed using boxplots. F = frontal, Sup = superior, Ant = anterior, Mid = middle, Med = medial, Inf = inferior, Orb = orbital, L = left, R = right. * < 0.05, ** < 0.01, *** < 0.001 after FDR correction for multiple comparisons; Cluster and connection characteristics are listed in Table S3.

The group comparison of FC between frontal regions connected via the aCC revealed several ROI-ROI connections with higher FC in the O-LP group compared to the O-HP group (Figs. 3 and 4, Table S3). These included both inter-hemispheric and intra-hemispheric frontal connections, which also include connections to parts of IFG, MFG, SFG, GR, and ACG. There were no ROI-ROI connections with significantly higher FC in the O-HP group. The connections that showed both a significant group difference in the comparison of FC between O-HP and O-LP and a significant correlation between FC and FA values in our second analysis were printed in bold in Tables S3 and S4.

### Correlation between FC and FA

**Fig. 5:**
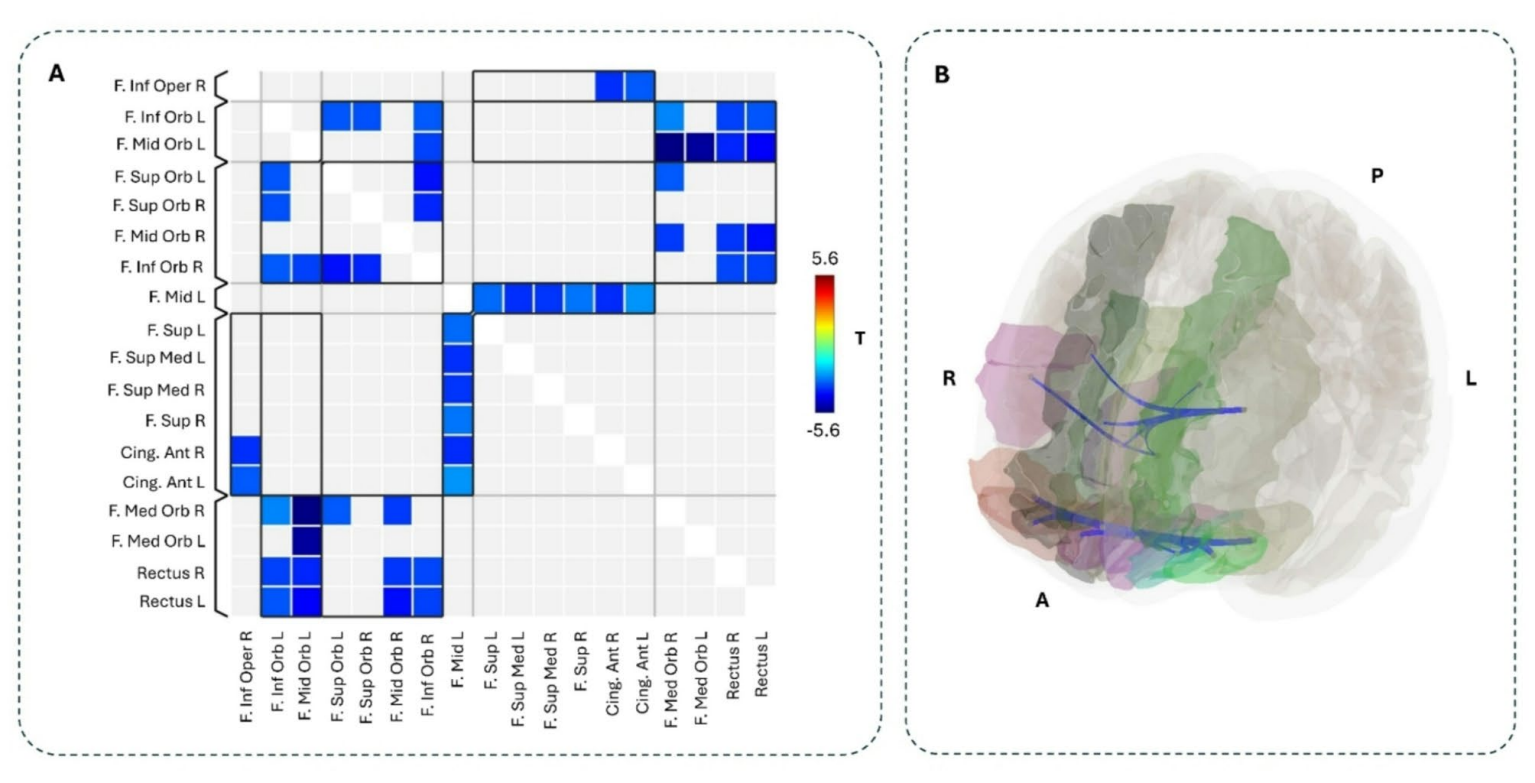
Correlation between FA and FC. **A** The colored matrix displays the T-values of significantly correlated ROI-ROI connections with FA values sorted along significant clusters from FNC statistics, blue: negatively correlated; **B** The ROIs, which show a significantly correlated connectivity with FA, are highlighted in different colors, connections that have a statistically significant correlation between FC and FA are shown in blue; R = Right, L = Left, A = Anterior, P = Posterior, F. = Frontal, Sup = Superior, Med = Medial, Mid = Middle, Orb = Orbital, Inf = Inferior, T = T-value.

**Fig. 6:**
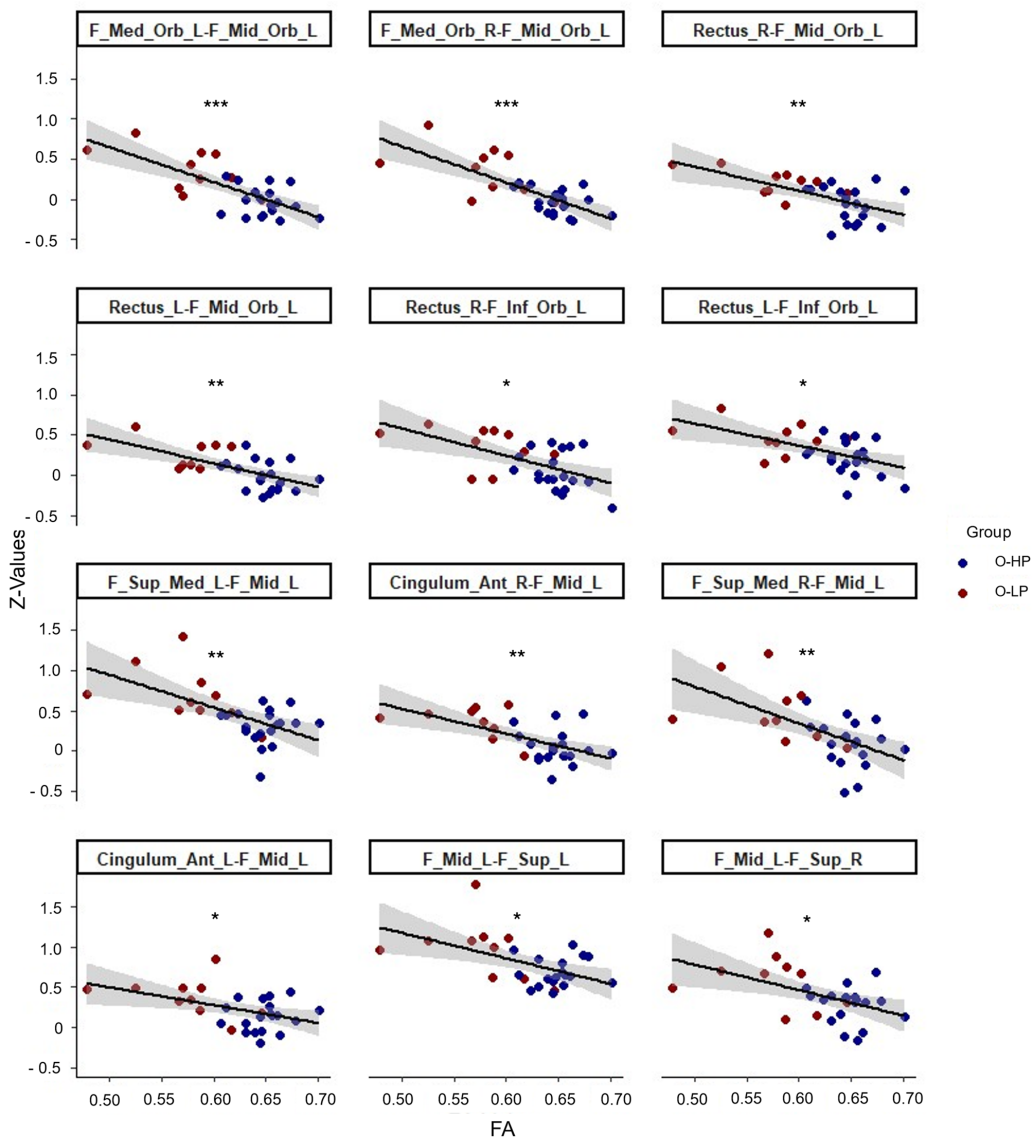
Correlation plots between FA-Values and Z-Values of the frontal connections, which exhibit both a significant group difference and a significant correlation with the FA values. F = frontal, Sup = superior, Ant = anterior, Mid = middle, Med = medial, Inf = inferior, Orb = orbital, L = left, R = right. * < 0.05, ** < 0.01, *** < 0.001 after FDR correction for multiple comparisons; Cluster and connection characteristics are listed in Table S4.

The correlation analyses between the FC values of the connections of the 22 selected ROIs and the individual mean FA values of the aCC across all 29 older participants showed negatively correlated ROI-ROI connections with the FA values (Figs. 5 and 6, Table S4). Here, as in the group comparison above, the negative correlation between FA and FC values included both intrahemispheric and interhemispheric connections, which also include connections to parts of the IFG, MFG, SFG, GR, and ACG. The connections that showed both a significant group difference in our first analyses and a significant correlation between FC and FA values in our second analyses were printed in bold in Tables S3 and S4. Due to the different group sizes, we have decided against an interaction analysis group*FA.

## Discussion

This study aimed to explore how degeneration of structural connectivity (SC) in older participants affects functional connectivity (FC) between connected brain regions and to examine whether groups of low- and high-performers in a complex tactile recognition task differ not only in SC but also in FC. Our primary finding is a negative correlation between FA in the aCC and FC between the connected regions across 29 healthy older participants. Second, older adults with low performance (O-LP) show significantly higher FC between several frontal ROIs compared to the higher performing group (O-HP). These findings are supported by the comparison of frontal FC between a younger group and O-LP, which revealed higher FC in O-LP across various frontal connections. A pattern that was not observed in the comparison between the younger group and O-HP.

Based on a hypothesis-driven approach, our analysis revealed a negative correlation between FC and microstructural characteristics (FA) of underlying structural pathways within frontal brain networks^59,60^. Several connections show both a negative correlation of FC with the FA values and a FC difference between the two older groups. The regions connected via the aCC include parts of the bilateral middle frontal gyrus (MFG), superior frontal gyrus (SFG), gyrus rectus (GR), and anterior part of the cingulate gyrus (ACG). These regions contribute to the prefrontal cortex which is implicated in various higher-order cognitive processes such as working memory^61^ and executive functions^62^ (dorso-lateral prefrontal cortex, DLPFC) and top-down processing of sensory information^63^ (ventro-lateral prefrontal cortex, VLPFC).

The whole-brain FC comparison between both older groups and the younger control group revealed several intra- and interhemispheric connections exhibiting significantly reduced FC in older participants and some connections showing significantly increased FC, primarily between posterior ROIs. Only O-LP demonstrated significantly increased FC between frontal ROIs relative to the younger group. The mixed pattern of both decreased and increased FC in older compared to younger participants aligns well with existing literature on healthy aging^22^.

At the group level, both older subgroups mainly showed reduced frontal FC compared to younger adults, consistent with age-related connectivity decline. However, the increase in FC observed in O-LP relative to O-HP may reflect a transient upregulation or dedifferentiation response specific to individuals with more pronounced structural degradation. Rather than being inconsistent, these patterns may represent distinct phases of age-related network reorganization, from early compensatory hyperconnectivity to eventual hypoconnectivity as reserve is exhausted.

Our previous work suggests that a decline in top-down processing due to decreased structural integrity of the connecting aCC may underlie reduced performance in complex tactile processing^34^. In this study, we demonstrate the possible functional consequences of this structural deterioration. We found that lower FA in the aCC correlates with higher FC between the connected regions. These opposing changes in FC may be explained by compensatory mechanisms that adapt neuronal processing to structural deterioration. The pattern is consistent with theoretical accounts of neural compensation, including the “Scaffolding Theory of Aging and Cognition” (STAC)^64^. The STAC model posits that age-related functional changes may reflect compensatory processes, where cognitive functions are preserved through the recruitment of additional brain regions or networks^65^. It has also been shown that healthy older adults with higher cognitive reserve levels exhibit increased activation in frontal regions. Cognitive reserve is thought to be a compensatory process that protects against cognitive impairment^66^. Our findings align with these hypotheses, suggesting that increased FC might represent a compensatory response to the degeneration of underlying structural pathways (i.e. the aCC), aimed at maintaining cognitive function^67,68^. We showed that participants with poor performance in the tactile recognition task exhibit reduced FA of the aCC, indicating compromised microstructural white matter characteristics. This decline could be an indication of advanced brain aging. As the brain’s structure deteriorates, it may trigger a compensatory increase in FC between prefrontal regions. This increased FC might help maintain normal performance in standard neuropsychological assessments but may not fully compensate for challenges in more complex tasks, such as tactile recognition. Therefore, poor performance in this task could be an early sign of cognitive decline, linked to structural damage that is not entirely offset by enhanced FC. Our results support the notion that an increase in FC occurs during the early stages of structural decline^30–32^. Few studies have combined structural and functional analyses in the early stages of age-related changes^69^. In task-related analysis, Hakun et al. found in a longitudinal study an increase in frontal task-dependent activation in healthy older adults associated with a decrease in FA in the anterior corpus callosum. The authors concluded that the increase in frontal activation may represent a mechanistic response to the reduced white matter integrity. These findings support our FC results. As structural disconnection progresses, these functional compensatory mechanisms may fail, leading to a decrease in FC, as observed in studies of advanced brain aging^22,25,27^.

Although the observed increase in frontal FC in lower-performing older adults may reflect compensatory recruitment of additional network resources, this interpretation remains provisional in the absence of direct brain–behavior correlations. Alternative explanations such as dedifferentiation or inefficient processing cannot be excluded. Our findings align with the broader conceptual framework of maintenance, reserve, and compensation in healthy aging^70^but further studies are needed to directly link FC alterations to behavioral outcomes.

We focused on frontal networks due to the observed isolated differences in SC in the aCC between O-LP and O-HP. However, the predominance of frontal functional alterations in older adults is consistent with previous research. Prior studies have demonstrated that aging induces changes in brain hemodynamics, particularly in frontal areas, without significant effects of gender or education^71^. The “posterior-anterior-shift in aging” (PASA) hypothesis^72^ suggests, that the frontal brain is more flexible in modulating functional connectivity with the hypothesis that the recruitment of frontal regions in older adults is a compensatory response to age-related cognitive decline^73^. The shift from a pronounced posterior to anterior brain region recruitment has been observed in both task-based fMRI^65^ and resting-state fMRI studies^72,74^. Although these changes are not yet clinically manifested, they are detectable through MRI scans. Our findings contribute to these hypotheses by adding a possible mechanistic basis with evidence for a compensatory upregulation of frontal FC due to age-related structural decline of the particularly vulnerable aCC. Our analysis focused on the interhemispheric aCC. Including additional intrahemispheric tracts in future work may help clarify whether FA–FC associations reflect general white matter degeneration or are specific to the aCC or interhemispheric disconnection.

There are several other limitations to note. First, the relatively small sample size of 29 older participants must be considered when interpreting the results. The correlation between FA and FC values, observed across all older adults, reflects a robust association with moderate to large effect sizes (*r* = − 0.45 to − 0.74). However, subgroup-specific analyses were not conducted due to limited power, particularly in the smaller O-LP group (*n* = 10), and the interpretation of these findings is restricted to the overall older cohort. Future studies should replicate these findings with larger cohorts and future studies on larger sample sizes are needed to verify the present results, directly test subgroup-specific associations and help to generalize the results to the broader population. Second, the design of this study was cross-sectional. A longitudinal approach could provide further insights into the temporal dynamics of FC and SC changes. Third, due to group allocation based on performance, the sample size of the two different groups, O-HP and O-LP differed, which may limit the statistical robustness of our first second-level analysis and is the reason why we did not perform an interaction analysis “group*FA”. Fourth, our findings should be correlated with detailed and sensitive neuropsychological testing to evaluate whether changes in SC and FC can be detected with relevant clinical tests beyond the observed decline in complex tactile pattern recognition. Fifth, FA values in the aCC for the young participants were not calculated in the previous study and could not be recalculated. Hence, a correlation with FC values was not possible.

## Conclusion

We found increased frontal functional connectivity in participants with poor tactile recognition performance. Increased FC was negatively correlated with the microstructural characteristics of underlying structural pathways in the whole group of older participants. This increase in frontal FC might represent a compensatory mechanism in response to structural decline aimed at maintaining cognitive function. Our findings suggest that frontal structural pathways, particularly the aCC, are most vulnerable to aging effects, leading to early structural decline and a subsequent focus on functional adaptations in frontal brain networks. As structural integrity further deteriorates, these compensatory mechanisms may eventually fail, resulting in the previously described decrease in FC with advanced age.

## Supplementary Information

Below is the link to the electronic supplementary material.

Supplementary Material 1

## Data Availability

The data will be made available upon reasonable request to the corresponding author, which includes submitting an analysis plan for a secondary project.

## References

[1] Jockwitz, C. & Caspers, S. Resting-state networks in the course of aging—differential insights from studies across the lifespan vs. amongst the old. *Pflüg Arch. - Eur. J. Physiol.* **473** (5), 793–803. 10.1007/s00424-021-02520-7 (2021). 10.1007/s00424-021-02520-7 PMC8076139 33576851

[2] Guo, Y. et al. Plasma proteomic profiles predict future dementia in healthy adults. *Nat. Aging*. **4** (2), 247–260. 10.1038/s43587-023-00565-0 (2024). 38347190 10.1038/s43587-023-00565-0

[3] Raz, N. & Rodrigue, K. M. Differential aging of the brain: patterns, cognitive correlates and modifiers. *Neurosci. Biobehav Rev.* **30** (6), 730–748. 10.1016/j.neubiorev.2006.07.001 (2006). 16919333 10.1016/j.neubiorev.2006.07.001 PMC6601348

[4] Smith, S. M. et al. Brain aging comprises many modes of structural and functional change with distinct genetic and biophysical associations. *eLife* **9**10.7554/eLife.52677 (2020). 10.7554/eLife.52677 PMC7162660 32134384

[5] Crous-Bou, M., Minguillón, C., Gramunt, N. & Molinuevo, J. L. Alzheimer’s disease prevention: from risk factors to early intervention. *Alzheimers Res. Ther.* **9** (1), 71. 10.1186/s13195-017-0297-z (2017). 28899416 10.1186/s13195-017-0297-z PMC5596480

[6] McDade, E., Llibre-Guerra, J. J., Holtzman, D. M., Morris, J. C. & Bateman, R. J. The informed road map to prevention of alzheimer disease: A call to arms. *Mol. Neurodegener*. **16** (1), 49. 10.1186/s13024-021-00467-y (2021). 34289882 10.1186/s13024-021-00467-y PMC8293489

[7] Higgen, F. L. et al. Congruency effects can compensate for deficits of healthy older adults in crossmodal integration. *BioRxiv Published Online June*. **19**, 673491. 10.1101/673491 (2019).

[8] Burzynska, A. Z. et al. Age-related differences in white matter microstructure: region-specific patterns of diffusivity. *NeuroImage* **49** (3), 2104–2112. 10.1016/j.neuroimage.2009.09.041 (2010). 19782758 10.1016/j.neuroimage.2009.09.041

[9] Damoiseaux, J. S. et al. White matter tract integrity in aging and alzheimer’s disease. *Hum. Brain Mapp.* **30** (4), 1051–1059. 10.1002/hbm.20563 (2009). 18412132 10.1002/hbm.20563 PMC6870688

[10] Schulz, R. et al. White matter integrity of motor connections related to training gains in healthy aging. *Neurobiol. Aging*. **35** (6), 1404–1411. 10.1016/j.neurobiolaging.2013.11.024 (2014). 24387983 10.1016/j.neurobiolaging.2013.11.024

[11] Deibert, E., Kraut, M., Kremen, S. & Hart, J. Neural pathways in tactile object recognition. *Neurology* **52** (7), 1413–1417. 10.1212/wnl.52.7.1413 (1999). 10227627 10.1212/wnl.52.7.1413

[12] Good, C. D. et al. A voxel-based morphometric study of ageing in 465 normal adult human brains. *NeuroImage* **14** (1 Pt 1), 21–36. 10.1006/nimg.2001.0786 (2001). 11525331 10.1006/nimg.2001.0786

[13] Jones, D. K., Knösche, T. R. & Turner, R. White matter integrity, fiber count, and other fallacies: the do’s and don’ts of diffusion MRI. *NeuroImage* **73**, 239–254. 10.1016/j.neuroimage.2012.06.081 (2013). 22846632 10.1016/j.neuroimage.2012.06.081

[14] Henschke, J. U., Ohl, F. W. & Budinger, E. Crossmodal connections of primary sensory cortices largely vanish during normal aging. *Front. Aging Neurosci.* **10**, 52. 10.3389/fnagi.2018.00052 (2018). 29551970 10.3389/fnagi.2018.00052 PMC5840148

[15] Kochunov, P. et al. Fractional anisotropy of water diffusion in cerebral white matter across the lifespan. *Neurobiol. Aging*. **33** (1), 9–20. 10.1016/j.neurobiolaging.2010.01.014 (2012). 20122755 10.1016/j.neurobiolaging.2010.01.014 PMC2906767

[16] Colangeli, S. et al. Cognitive reserve in healthy aging and alzheimer’s disease: A Meta-Analysis of fMRI studies. *Am. J. Alzheimers Dis. Dementiasr*. **31** (5), 443–449. 10.1177/1533317516653826 (2016). 10.1177/1533317516653826 PMC10852844 27307143

[17] Oghabian, M. A., Batouli, S. A. H., Norouzian, M., Ziaei, M. & Sikaroodi, H. Using functional magnetic resonance imaging to differentiate between healthy aging subjects, mild cognitive impairment, and alzheimer’s patients. *J Res. Med. Sci*. **15**(2), 84–93 (2009). PMC3082789 21526064

[18] Kasper, J. et al. Resting state changes in aging and parkinson’s disease are shaped by underlying neurotransmission – a normative modeling study. *Biol psychiatry Cogn neurosci neuroimaging*. *Published Online April* **2024**:S2451902224001125. 10.1016/j.bpsc.2024.04.010 10.1016/j.bpsc.2024.04.010 38679325

[19] Madden, D. J., Bennett, I. J. & Song, A. W. Cerebral white matter integrity and cognitive aging: contributions from diffusion tensor imaging. *Neuropsychol. Rev.* **19** (4), 415–435. 10.1007/s11065-009-9113-2 (2009). 19705281 10.1007/s11065-009-9113-2 PMC2787975

[20] Hagmann, P. et al. Mapping the structural core of human cerebral cortex. *PLoS Biol.* **6** (7), e159. 10.1371/journal.pbio.0060159 (2008). 18597554 10.1371/journal.pbio.0060159 PMC2443193

[21] Skudlarski, P. et al. Measuring brain connectivity: diffusion tensor imaging validates resting state Temporal correlations. *NeuroImage* **43** (3), 554–561. 10.1016/j.neuroimage.2008.07.063 (2008). 18771736 10.1016/j.neuroimage.2008.07.063 PMC4361080

[22] Fjell, A. M. et al. Relationship between structural and functional connectivity change across the adult lifespan: A longitudinal investigation. *Hum. Brain Mapp.* **38** (1), 561–573. 10.1002/hbm.23403 (2017). 27654880 10.1002/hbm.23403 PMC5148650

[23] Hirsiger, S. et al. Structural and functional connectivity in healthy aging: associations for cognition and motor behavior. *Hum. Brain Mapp.* **37** (3), 855–867. 10.1002/hbm.23067 (2015). 26663386 10.1002/hbm.23067 PMC6867683

[24] Tsang, A. et al. White matter structural connectivity is not correlated to cortical Resting-State functional connectivity over the healthy adult lifespan. *Front. Aging Neurosci.* **9**, 144. 10.3389/fnagi.2017.00144 (2017). 28572765 10.3389/fnagi.2017.00144 PMC5435815

[25] Hinault, T., Larcher, K., Bherer, L., Courtney, S. M. & Dagher, A. Age-related differences in the structural and effective connectivity of cognitive control: a combined fMRI and DTI study of mental arithmetic. *Neurobiol. Aging*. **82**, 30–39. 10.1016/j.neurobiolaging.2019.06.013 (2019). 31377538 10.1016/j.neurobiolaging.2019.06.013

[26] Lynn, C. W. & Bassett, D. S. The physics of brain network structure, function, and control. *Nat. Rev. Phys.* **1** (5), 318–332. 10.1038/s42254-019-0040-8 (2019).

[27] Suárez, L. E., Markello, R. D., Betzel, R. F. & Misic, B. Linking structure and function in macroscale brain networks. *Trends Cogn. Sci.* **24** (4), 302–315. 10.1016/j.tics.2020.01.008 (2020). 32160567 10.1016/j.tics.2020.01.008

[28] Damoiseaux, J. S. et al. Consistent resting-state networks across healthy subjects. *Proc. Natl. Acad. Sci. U S A*. **103** (37), 13848–13853. 10.1073/pnas.0601417103 (2006). 16945915 10.1073/pnas.0601417103 PMC1564249

[29] van den Heuvel, M. P., Stam, C. J., Boersma, M. & Hulshoff Pol, H. E. Small-world and scale-free organization of voxel-based resting-state functional connectivity in the human brain. *NeuroImage* **43** (3), 528–539. 10.1016/j.neuroimage.2008.08.010 (2008). 18786642 10.1016/j.neuroimage.2008.08.010

[30] Stumme, J. et al. Interrelating differences in structural and functional connectivity in the older adult’s brain. *Hum. Brain Mapp.* **43** (18), 5543–5561. 10.1002/hbm.26030 (2022). 35916531 10.1002/hbm.26030 PMC9704795

[31] Bai, F. et al. Specifically progressive deficits of brain functional marker in amnestic type mild cognitive impairment. *PloS One*. **6** (9), e24271. 10.1371/journal.pone.0024271 (2011). 21935394 10.1371/journal.pone.0024271 PMC3174167

[32] Damoiseaux, J. S., Prater, K. E., Miller, B. L. & Greicius, M. D. Functional connectivity tracks clinical deterioration in alzheimer’s disease. *Neurobiol. Aging*. **33** (4), 828e19–828e30. 10.1016/j.neurobiolaging.2011.06.024 (2012). 10.1016/j.neurobiolaging.2011.06.024 PMC3218226 21840627

[33] Hawellek, D. J., Hipp, J. F., Lewis, C. M., Corbetta, M. & Engel, A. K. Increased functional connectivity indicates the severity of cognitive impairment in multiple sclerosis. *Proc. Natl. Acad. Sci. U S A*. **108** (47), 19066–19071. 10.1073/pnas.1110024108 (2011). 22065778 10.1073/pnas.1110024108 PMC3223469

[34] Higgen, F. L. et al. Reduced frontal white matter microstructure in healthy older adults with low tactile recognition performance. *Sci. Rep.* **11** (1), 1–13. 10.1038/s41598-021-90995-w (2021). 34083614 10.1038/s41598-021-90995-w PMC8175740

[35] Freiherr, J., Lundström, J. N., Habel, U. & Reetz, K. Multisensory integration mechanisms during aging. *Front. Hum. Neurosci.* **7**, 863. 10.3389/fnhum.2013.00863 (2013). 24379773 10.3389/fnhum.2013.00863 PMC3861780

[36] Davis, A. et al. Aging and hearing health: the Life-course approach. *Gerontologist* **56** (Suppl_2), S256–S267. 10.1093/geront/gnw033 (2016). 26994265 10.1093/geront/gnw033 PMC6283365

[37] Kenshalo, D. R. Somesthetic sensitivity in young and elderly humans. *J. Gerontol.* **41** (6), 732–742. 10.1093/geronj/41.6.732 (1986). 3772049 10.1093/geronj/41.6.732

[38] Kalina, R. E. Seeing into the future: vision and aging. *West. J. Med.* **167** (4), 253–257 (1997). 9348756 PMC1304540

[39] Lindenberger, U. & Baltes, P. B. Sensory Functioning and Intelligence in Old Age: A Strong Connection. 10.1037//0882-7974.9.3.339 7999320

[40] Tay, T. et al. Sensory and cognitive association in older persons: findings from an older Australian population. *Gerontology* **52** (6), 386–394. 10.1159/000095129 (2006). 16921251 10.1159/000095129

[41] Schubert, C. R. et al. Sensory impairments and cognitive function in Middle-Aged adults. *J. Gerontol. Ser. A*. **72** (8), 1087–1090. 10.1093/gerona/glx067 (2017). 10.1093/gerona/glx067 PMC5861860 28535277

[42] Humes, L. E. & Young, L. A. Sensory–Cognitive interactions in older adults. *Ear Hear.* **37** (1), 52S–61S. 10.1097/AUD.0000000000000303 (2016). 27355770 10.1097/AUD.0000000000000303 PMC4930008

[43] Misselhorn, J. et al. Sensory capability and information integration independently explain the cognitive status of healthy older adults. *Sci. Rep.* **10** (1), 22437. 10.1038/s41598-020-80069-8 (2020). 33384454 10.1038/s41598-020-80069-8 PMC7775431

[44] Oldfield, R. C. The assessment and analysis of handedness: the Edinburgh inventory. *Neuropsychologia* **9** (1), 97–113. 10.1016/0028-3932(71)90067-4 (1971). 5146491 10.1016/0028-3932(71)90067-4

[45] Folstein, M. F., Folstein, S. E. & McHugh, P. R. Mini-mental state. A practical method for grading the cognitive state of patients for the clinician. *J. Psychiatr Res.* **12** (3), 189–198. 10.1016/0022-3956(75)90026-6 (1975). 1202204 10.1016/0022-3956(75)90026-6

[46] Kalbe, E. et al. DemTect: a new, sensitive cognitive screening test to support the diagnosis of mild cognitive impairment and early dementia. *Int. J. Geriatr. Psychiatry*. **19** (2), 136–143. 10.1002/gps.1042 (2004). 14758579 10.1002/gps.1042

[47] Crosby, P. M. & Dellon, A. L. Comparison of two-point discrimination testing devices. *Microsurgery* **10** (2), 134–137 (1989). 2770513 10.1002/micr.1920100214

[48] Fruhstorfer, H., Gross, W. & Selbmann, O. Von Frey hairs: new materials for a new design. *Eur. J. Pain Lond. Engl.* **5** (3), 341–342. 10.1053/eujp.2001.0250 (2001). 10.1053/eujp.2001.0250 11558991

[49] Zimmermann, P., Messner, C., Poser, U. & Sedelmeier, P. Ein Fragebogen erlebter defizite der aufmerksamkeit (FEDA) [A questionnaire measuring Self-Experienced deficits of attention]. *Psychol Univ. Freibg Freibg* Published online (1991).

[50] Whitfield-gabrieli, S., Nieto-castanon, A. & Conn A functional connectivity toolbox for correlated and anticorrelated brain networks. *Brain Connect.* **2** (3). 10.1089/brain.2012.0073 (2012). 10.1089/brain.2012.0073 22642651

[51] Braaß, H. et al. Early functional connectivity alterations in contralesional motor networks influence outcome after severe stroke. *Sci. Rep.* **13**, 1–8. 10.1038/s41598-023-38066-0 (2023). 37419966 10.1038/s41598-023-38066-0 PMC10328915

[52] Behzadi, Y., Restom, K., Liau, J. & Liu, T. T. A component based noise correction method (CompCor) for BOLD and perfusion based fMRI. *NeuroImage* **37** (1), 90–101. 10.1016/j.neuroimage.2007.04.042 (2007). 17560126 10.1016/j.neuroimage.2007.04.042 PMC2214855

[53] Murphy, K., Birn, R. M., Handwerker, D. A., Jones, T. B. & Bandettini, P. A. The impact of global signal regression on resting state correlations: are anti-correlated networks introduced? *NeuroImage* **44** (3), 893–905. 10.1016/j.neuroimage.2008.09.036 (2009). 18976716 10.1016/j.neuroimage.2008.09.036 PMC2750906

[54] Hallquist, M. N., Hwang, K. & Luna, B. The nuisance of nuisance regression: spectral misspecitifaction in a common approach to Resting-State fMRI preprocessing reintroduces noise an obscures functional connectivity. *NeuroImage* **82**, 208–225. 10.1038/jid.2014.371 (2013). 23747457 10.1016/j.neuroimage.2013.05.116 PMC3759585

[55] Tzourio-Mazoyer, N. et al. Automated anatomical labeling of activations in SPM using a macroscopic anatomical parcellation of the MNI MRI single-subject brain. *NeuroImage* **15** (1), 273–289. 10.1006/nimg.2001.0978 (2002). 11771995 10.1006/nimg.2001.0978

[56] Rüsch, N. et al. Reduced interhemispheric structural connectivity between anterior cingulate cortices in borderline personality disorder. *Psychiatry Res. Neuroimaging*. **181** (2), 151–154. 10.1016/j.pscychresns.2009.08.004 (2010). 10.1016/j.pscychresns.2009.08.004 20079614

[57] Park, H. et al. Corpus callosal connection mapping using cortical Gray matter parcellation and DT-MRI. *Hum. Brain Mapp.* **29** (5), 503–516. 10.1002/hbm.20314 (2008). 17133394 10.1002/hbm.20314 PMC6870924

[58] Jafri, M. J., Pearlson, G. D., Stevens, M. & Calhoun, V. D. A method for functional network connectivity among spatially independent resting-state components in schizophrenia. *NeuroImage* **39** (4), 1666–1681. 10.1016/j.neuroimage.2007.11.001 (2008). 18082428 10.1016/j.neuroimage.2007.11.001 PMC3164840

[59] Fabri, M., Pierpaoli, C., Barbaresi, P. & Polonara, G. Functional topography of the corpus callosum investigated by DTI and fMRI. *World J. Radiol.* **6** (12). 10.4329/wjr.v6.i12.895 (2014). 10.4329/wjr.v6.i12.895 PMC4278150 25550994

[60] Hofer, S. & Frahm, J. Topography of the human corpus callosum revisited—Comprehensive fiber tractography using diffusion tensor magnetic resonance imaging. *NeuroImage* **32** (3), 989–994. 10.1016/j.neuroimage.2006.05.044 (2006). 16854598 10.1016/j.neuroimage.2006.05.044

[61] Lara, A. H. & Wallis, J. D. The role of prefrontal cortex in working memory: A mini review. *Front. Syst. Neurosci.* **9**10.3389/fnsys.2015.00173 (2015). 10.3389/fnsys.2015.00173 PMC4683174 26733825

[62] Gläscher, J. et al. Lesion mapping of cognitive control and value-based decision making in the prefrontal cortex. *Proc. Natl. Acad. Sci.* **109** (36), 14681–14686. 10.1073/pnas.1206608109 (2012). 22908286 10.1073/pnas.1206608109 PMC3437894

[63] Uno, T. et al. Dissociated roles of the inferior frontal gyrus and superior Temporal sulcus in audiovisual processing: Top-Down and Bottom-Up mismatch detection. *PLoS ONE*. **10** (3). 10.1371/journal.pone.0122580 (2015). 10.1371/journal.pone.0122580 PMC4379108 25822912

[64] Park, D. C. & Reuter-Lorenz, P. The adaptive brain: aging and neurocognitive scaffolding. *Annu. Rev. Psychol.* **60** (1), 173–196. 10.1146/annurev.psych.59.103006.093656 (2009). 19035823 10.1146/annurev.psych.59.103006.093656 PMC3359129

[65] Ren, P., Anthony, M., Aarsland, D., Wu, D. & Commentary A posterior-to-anterior shift of brain functional dynamics in aging. *Front. Aging Neurosci.* **11**, 341. 10.3389/fnagi.2019.00341 (2019). 31920623 10.3389/fnagi.2019.00341 PMC6916628

[66] Varela-López, B. et al. Cognitive reserve, neurocognitive performance, and high-order resting-state networks in cognitively unimpaired aging. *Neurobiol. Aging*. **117**, 151–164. 10.1016/j.neurobiolaging.2022.05.012 (2022). 35759984 10.1016/j.neurobiolaging.2022.05.012

[67] Farras-Permanyer, L. et al. Age-related changes in resting-state functional connectivity in older adults. *Neural Regen Res.* **14** (9), 1544. 10.4103/1673-5374.255976 (2019). 31089053 10.4103/1673-5374.255976 PMC6557095

[68] Seidler, R. D. et al. Motor control and aging: links to age-related brain structural, functional, and biochemical effects. *Neurosci. Biobehav Rev.* **34** (5), 721–733. 10.1016/j.neubiorev.2009.10.005 (2010). 19850077 10.1016/j.neubiorev.2009.10.005 PMC2838968

[69] Betzel, R. F. et al. Changes in structural and functional connectivity among resting-state networks across the human lifespan. *NeuroImage Published Online August*. 10.1016/j.neuroimage.2014.07.067 (2014). 10.1016/j.neuroimage.2014.07.067 25109530

[70] Cabeza, R. et al. Maintenance, reserve and compensation: the cognitive neuroscience of healthy ageing. *Nat. Rev. Neurosci.* **19** (11), 701–710. 10.1038/s41583-018-0068-2 (2018). 30305711 10.1038/s41583-018-0068-2 PMC6472256

[71] Kavroulakis, E. et al. Evidence of Age-Related hemodynamic and functional connectivity impairment: A resting state fMRI study. *Front. Neurol.* **12**, 633500. 10.3389/fneur.2021.633500 (2021). 33833727 10.3389/fneur.2021.633500 PMC8021915

[72] McCarthy, P., Benuskova, L. & Franz, E. A. The age-related posterior-anterior shift as revealed by Voxelwise analysis of functional brain networks. *Front. Aging Neurosci.* **6**10.3389/fnagi.2014.00301 (2014). 10.3389/fnagi.2014.00301 PMC4224138 25426065

[73] Stern, Y. What is cognitive reserve? Theory and research application of the reserve concept. *J. Int. Neuropsychol. Soc. JINS*. **8** (3), 448–460 (2002). 11939702

[74] Zhang, H., Lee, A. & Qiu, A. A posterior-to-anterior shift of brain functional dynamics in aging. *Brain Struct. Funct.* **222** (8), 3665–3676. 10.1007/s00429-017-1425-z (2017). 28417233 10.1007/s00429-017-1425-z

