# Correction: Increased frontal functional connectivity correlates with structural disconnection in the anterior corpus callosum in healthy older adults

**DOI:** 10.1038/s41598-025-31542-9

**Published:** 2025-12-19

**Authors:** Hanna Braaß, Nadine Reiter, Winifried Backhaus, Paweł P. Wróbel, Fanny Quandt, Robert Schulz, Christian Gerloff, Focko L. Higgen

**Affiliations:** 1 Department of Neurology, University Medical Center Hamburg-Eppendorf, Martinistraße 52, 20246 Hamburg, Germany; 2 Department of Systems Neuroscience, University Medical Center Hamburg- Eppendorf, Martinistraße 52, 20246 Hamburg, Germany; 3 Department of Psychiatry and Psychotherapy, University Medical Center Hamburg- Eppendorf, Martinistraße 52, 20246 Hamburg, Germany

Correction to: *Scientific Reports* 10.1038/s41598-025-19143-y, published online 12 September 2025.

The original version of this Article contained errors.

The legends of Figures 1, 2, 3 and 5 were erroneously added in the body of the text.

In addition, in the Results section, under the subheading ‘Assessment’, the text was interchanged with the legend of Table 1. As a result,

“The assessment data of the groups of included younger (= Y) and older participants (= O) is shown and additionally divided into two groups, older-low-performers (= O-P) and older-high-performers (= O-HP). Mean values are shown ± standard deviation. *The mean FA-value in the aCC showed a significant difference between O-LP and O-HP (two-sided unpaired t-test, *p* < 0.001). The table is a modified version of a table in^34^. ^+^Indicate significant differences between Young and O-LP (*p* values  ≤  0.01). ^#^Indicate significant differences between Young and O-HP (*p* values  ≤  0.01). No significant behavioral differences between O-LP and O-HP were observed except for FA. Mini-Mental State (MMSE), mechanical detection threshold (MDT), “Fragebogen erlebter Defizite der Aufmerksamkeit” (FEDA), fractional anisotropy (FA).”

now reads:

“Table 1 presents the results of the clinical and behavioral assessment of the different groups and the mean FA in the aCC of the older participants. There was a significant difference in FA in the aCC between O-LP and O-HP, but no significant differences in the assessments.”

The original Article has been corrected.

