# Supplementary material for: Increased frontal functional connectivity correlates with structural disconnection in the anterior corpus callosum in healthy older adults
Source: Sci Rep. 2025 Sep 12;15:32489. doi: 10.1038/s41598-025-19143-y (PMC12432219; doi:10.1038/s41598-025-19143-y)
Supplement: Supplementary file 1 — Supplementary Material 1 [file 41598_2025_19143_MOESM1_ESM.docx]

**Supplementary Materials**

**Increased frontal functional connectivity correlates with structural disconnection in the anterior corpus callosum in healthy older adults**

Hanna Braaß^1,2*#^, Nadine Reiter^1^*, Winifried Backhaus^1^, Paweł P. Wróbel ^1^, Fanny Quandt^1^, Robert Schulz^1^, Christian Gerloff^1^, Focko L. Higgen^1,3^

^1^ Department of Neurology, University Medical Center Hamburg-Eppendorf, Martinistraße 52, 20246 Hamburg, Germany.

^2^ Department of Systems Neuroscience, University Medical Center Hamburg-Eppendorf, Martinistraße 52, 20246 Hamburg, Germany.

^3^ Department of Psychiatry and Psychotherapy, University Medical Center Hamburg-Eppendorf, Martinistraße 52, 20246 Hamburg, Germany.

*These authors contributed equally: Hanna Braaß and Nadine Reiter

| Nr | **Side** | **Label** | **Anatomical** |
| --- | --- | --- | --- |
| 1 | L | Frontal Sup | Superior frontal gyrus, dorsolateral |
| 2 | R | Frontal Sup | Superior frontal gyrus, dorsolateral |
| 3 | L | Frontal Sup Orb | Superior frontal gyrus, orbital |
| 4 | R | Frontal Sup Orb | Superior frontal gyrus, orbital |
| 5 | L | Frontal Mid | Middle frontal gyrus |
| 6 | R | Frontal Mid | Middle frontal gyrus |
| 7 | L | Frontal Mid Orb | Middle frontal gyrus, orbital |
| 8 | R | Frontal Mid Orb | Middle frontal gyrus, orbital |
| 9 | L | Frontal Inf Oper | Inferior frontal gyrus, opercular |
| 10 | R | Frontal Inf Oper | Inferior frontal gyrus, opercular |
| 11 | L | Frontal Inf Tri | Inferior frontal gyrus, triangular |
| 12 | R | Frontal Inf Tri | Inferior frontal gyrus, triangular |
| 13 | L | Frontal Inf Orb | Inferior frontal gyrus, orbital |
| 14 | R | Frontal Inf Orb | Inferior frontal gyrus, orbital |
| 15 | L | Frontal Sup Med | Superior frontal gyrus, medial |
| 16 | R | Frontal Sup Med | Superior frontal gyrus, medial |
| 17 | L | Frontal Med Orb | Superior frontal gyrus, medial orbital |
| 18 | R | Frontal Med Orb | Superior frontal gyrus, medial orbital |
| 19 | L | Rectus | Gyrus rectus |
| 20 | R | Rectus | Gyrus rectus |
| 21 | L | Cingulum Ant | Cingulate gyrus, anterior part |
| 22 | R | Cingulum Ant | Cingulate gyrus, anterior part |

**Table S1** | List of the AAL ROIs used

| **Cluster 1: F(2,27) = 9.38, p-unc = 0.0008, p-FDR = 0.0136** | | | | | | | **p-FDR** | |
| --- | --- | --- | --- | --- | --- | --- | --- | --- |
| Frontal Inf Orb R | - | Frontal Inf Oper R | T(28) | = | -3.26 | 0.02 | |  |
| **Cluster 2: F(2,27) = 8.51, p-unc = 0.001, p-FDR = 0.0136** | | | | | | | **p-FDR** | |
| **Frontal Inf Orb L** | **-** | **Rectus L** | **T(28)** | **=** | **4.35** | **0.003** | |  |
| **Frontal Inf Orb L** | **-** | **Rectus R** | **T(28)** | **=** | **4.13** | **0.003** | |  |
| Frontal Inf Orb L | - | Frontal Med Orb R | T(28) | = | 2.98 | 0.04 | |  |
| Frontal Inf Orb L | - | Frontal Med Orb L | T(28) | = | 2.88 | 0.04 | |  |
| **Cluster 3: F(2,27) = 7.76, p-unc = 0.002, p-FDR = 0.0145** | | | | | | | **p-FDR** | |
| Frontal Inf Orb R | - | Rectus R | T(28) | = | 3.42 | 0.02 | |  |
| Frontal Inf Orb R | - | Rectus L | T(28) | = | 3.41 | 0.02 | |  |
| Frontal Inf Orb R | - | Frontal Med Orb L | T(28) | = | 3.19 | 0.02 | |  |
| **Cluster 4: F(2,27) = 7.14, p-unc = 0.003, p-FDR = 0.016** | | | | | | | **p-FDR** | |
| **Frontal Mid L** | **-** | **Frontal Sup Med L** | **T(28)** | **=** | **3.85** | **0.007** | |  |
| **Frontal Mid L** | **-** | **Frontal Sup Med R** | **T(28)** | **=** | **3.82** | **0.007** | |  |
| **Frontal Mid L** | **-** | **Frontal Sup R** | **T(28)** | **=** | **2.72** | **0.047** | |  |
| **Frontal Mid L** | **-** | **Cingulum Ant R** | **T(28)** | **=** | **2.72** | **0.047** | |  |
| **Cluster 5: F(2,27) = 6.82, p-unc = 0.004, p-FDR = 0.016** | | | | | | | **p-FDR** | |
| Frontal Sup L | - | Frontal Sup R | T(28) | = | 5.33 | 0.0002 | |  |
| Frontal Sup L | - | Frontal Sup Med R | T(28) | = | 4.18 | 0.003 | |  |
| Frontal Sup L | - | Cingulum Ant R | T(28) | = | 3.61 | 0.008 | |  |
| Frontal Sup Med L | - | Frontal Sup Med R | T(28) | = | 3.05 | 0.03 | |  |
| Frontal Sup Med L | - | Cingulum Ant R | T(28) | = | 2.97 | 0.03 | |  |
| Frontal Sup Med L | - | Frontal Sup R | T(28) | = | 2.64 | 0.04 | |  |
| **Cluster 6: F(2,27) = 6.39, p-unc = 0.005, p-FDR = 0.018** | | | | | | | **p-FDR** | |
| Frontal Inf Orb R | - | Frontal Sup Orb L | T(28) | = | 3.1 | 0.02 | |  |
| Frontal Inf Orb R | - | Frontal Sup Orb R | T(28) | = | 2.67 | 0.04 | |  |
|  |  |  |  |  |  |  | |  |

**Table S2 | Significant clusters of different FC between the two groups, FC(O-LP) > FC(Y)**

Cluster results and specific results for significant differences between the FC values of the two groups. Connection threshold p < 0.05 (FDR corrected), cluster threshold p < 0.05 (FDR corrected). Connections that have significantly higher FC values in the O-LP group in comparison with Y and in comparison with O-HP are highlighted in bold.

| **Cluster 1: F(2,26) = 8.94, p-unc = 0.001, p-FDR = 0.023** | | | | | | **p-FDR** |
| --- | --- | --- | --- | --- | --- | --- |
| **Frontal Mid Orb L** | **-** | **Frontal Med Orb L** | **T(27)** | **=** | **4.74** | **0.0008** |
| **Frontal Mid Orb L** | **-** | **Frontal Med Orb R** | **T(27)** | **=** | **4.65** | **0.0008** |
| **Frontal Mid Orb L** | **-** | **Rectus R** | **T(27)** | **=** | **3.56** | **0.008** |
| **Frontal Mid Orb L** | **-** | **Rectus L** | **T(27)** | **=** | **3.55** | **0.008** |
| Frontal Inf Tri L | - | Frontal Med Orb L | T(27) | = | 3.52 | 0.02 |
| **Frontal Inf Orb L** | **-** | **Rectus R** | **T(27)** | **=** | **3.26** | **0.03** |
| **Frontal Inf Orb L** | **-** | **Rectus L** | **T(27)** | **=** | **3.01** | **0.04** |
| **Cluster 2: F(2,26) = 7.07, p-unc = 0.004, p-FDR = 0.037** | | | | | | **p-FDR** |
| **Frontal Mid L** | **-** | **Frontal Sup Med L** | **T(27)** | **=** | **3.7** | **0.02** |
| **Frontal Mid L** | **-** | **Cingulum Ant R** | **T(27)** | **=** | **3.39** | **0.02** |
| **Frontal Mid L** | **-** | **Frontal Sup Med R** | **T(27)** | **=** | **3.2** | **0.02** |
| **Frontal Mid L** | **-** | **Cingulum Ant L** | **T(27)** | **=** | **3.11** | **0.02** |
| **Frontal Mid L** | **-** | **Frontal Sup L** | **T(27)** | **=** | **3.04** | **0.02** |
| **Frontal Mid L** | **-** | **Frontal Sup R** | **T(27)** | **=** | **2.99** | **0.02** |
| Frontal Mid R | - | Frontal Sup Med L | T(27) | = | 3.3 | 0.03 |
| Frontal Mid R | - | Frontal Sup Med R | T(27) | = | 3.1 | 0.03 |
| Frontal Mid R | - | Cingulum Ant L | T(27) | = | 2.87 | 0.04 |

**Table S3 | Significant clusters of different FC between the two groups, FC(O-LP) > FC(O-HP)**

Cluster results and specific results for significant differences between the FC values of the two groups. Connection threshold p < 0.05 (FDR corrected), cluster threshold p < 0.05 (FDR corrected). Connections that have significantly higher FC values in the O-LP group and are identical to connections that correlate significantly with FA values (Table S3) are highlighted in bold.

| **Cluster 1 : F(2,26) = 11.28, p-unc = 0.0003, p-FDR = 0.006** | | | | | | **p-FDR** |
| --- | --- | --- | --- | --- | --- | --- |
| **Frontal Mid Orb L** | **-** | **Frontal Med Orb R** | **T(27)** | **=** | **-5.65** | **0.0001** |
| **Frontal Mid Orb L** | **-** | **Frontal Med Orb L** | **T(27)** | **=** | **-5.35** | **0.0001** |
| **Frontal Mid Orb L** | **-** | **Rectus L** | **T(27)** | **=** | **-4.29** | **0.001** |
| **Frontal Mid Orb L** | **-** | **Rectus R** | **T(27)** | **=** | **-3.85** | **0.003** |
| **Frontal Inf Orb L** | **-** | **Rectus R** | **T(27)** | **=** | **-3.57** | **0.01** |
| **Frontal Inf Orb L** | **-** | **Rectus L** | **T(27)** | **=** | **-3.37** | **0.01** |
| Frontal Inf Orb L | - | Frontal Med Orb R | T(27) | = | -2.79 | 0.03 |
| **Cluster 2 : F(2,26) = 9.57, p-unc = 0.0008, p-FDR = 0.008** | | | | | | **p-FDR** |
| Frontal Sup Orb L | - | Frontal Inf Orb R | T(27) | = | -4.14 | 0.006 |
| Frontal Sup Orb R | - | Frontal Inf Orb R | T(27) | = | -3.9 | 0.01 |
| **Cluster 3 : F(2,26) = 8.68, p-unc = 0.001, p-FDR = 0.009** | | | | | | **p-FDR** |
| Frontal Mid Orb R | - | Rectus L | T(27) | = | -4.14 | 0.006 |
| Frontal Inf Orb R | - | Rectus L | T(27) | = | -3.56 | 0.006 |
| Frontal Inf Orb R | - | Rectus R | T(27) | = | -3.53 | 0.006 |
| Frontal Mid Orb R | - | Rectus R | T(27) | = | -3.69 | 0.008 |
| Frontal Mid Orb R | - | Frontal Med Orb R | T(27) | = | -3.65 | 0.008 |
| Frontal Sup Orb L | - | Frontal Med Orb R | T(27) | = | -3.27 | 0.02 |
| **Cluster 4 : F(2,26) = 6.36, p-unc = 0.006, p-FDR = 0.03** | | | | | | **p-FDR** |
| **Frontal Mid L** | **-** | **Cingulum Ant R** | **T(27)** | **=** | **-3.81** | **0.007** |
| **Frontal Mid L** | **-** | **Frontal Sup Med L** | **T(27)** | **=** | **-3.75** | **0.007** |
| **Frontal Mid L** | **-** | **Frontal Sup Med R** | **T(27)** | **=** | **-3.69** | **0.007** |
| **Frontal Mid L** | **-** | **Frontal Sup L** | **T(27)** | **=** | **-3.1** | **0.02** |
| **Frontal Mid L** | **-** | **Frontal Sup R** | **T(27)** | **=** | **-3.01** | **0.02** |
| **Frontal Mid L** | **-** | **Cingulum Ant L** | **T(27)** | **=** | **-2.63** | **0.048** |
| **Cluster 5 : F(2,26) = 5.19, p-unc = 0.01, p-FDR = 0.049** | | | | | | **p-FDR** |
| Frontal Mid Orb L | - | Frontal Inf Orb R | T(27) | = | -3.57 | 0.006 |
| Frontal Inf Orb L | - | Frontal Sup Orb R | T(27) | = | -3.41 | 0.01 |
| Frontal Inf Orb L | - | Frontal Sup Orb L | T(27) | = | -3.34 | 0.01 |
| Frontal Inf Orb L | - | Frontal Inf Orb R | T(27) | = | -3.31 | 0.01 |
| **Cluster 7 : F(2,26) = 4.83, p-unc = 0.016, p-FDR = 0.049** | | | | | | **p-FDR** |
| Frontal Inf Oper R | - | Cingulum Ant R | T(27) | = | -3.77 | 0.02 |
| Frontal Inf Oper R | - | Cingulum Ant L | T(27) | = | -3.29 | 0.03 |

**Table S4 | Significant clusters for the correlation between FA and FC**

Cluster results and specific results for significant ROI-ROI connections are presented. Connection threshold p < 0.05 (FDR corrected), cluster threshold p < 0.05 (FDR corrected). Cluster 6 is not presented because the included connections did not survive FDR correction. Connections that have significantly higher FC values in the O-LP group (Table 2) and are identical to connections that correlate significantly with FA values are highlighted in bold.
